# A stochastic model for early placental development^†^

**DOI:** 10.1098/rsif.2014.0149

**Published:** 2014-08-06

**Authors:** Simon L. Cotter, Václav Klika, Laura Kimpton, Sally Collins, Alexander E. P. Heazell

**Affiliations:** 1 School of Mathematics, University of Manchester, Oxford Road, Manchester, UK; 2 Department of Mathematics, FNSPE, Czech Technical University in Prague, Trojanova 13, Prague 2 12000, Czech Republic; 3 Mathematical Institute, University of Oxford, Woodstock Road, Oxford, UK; 4 Nuffield Department of Obstetrics and Gynaecology, University of Oxford, Oxford, UK; 5 Fetal Medicine Unit, John Radcliffe Hospital, Oxford, UK; 6 Institute of Human Development, Maternal and Fetal Health Research Centre, University of Manchester, Manchester, UK; 7 Maternal and Fetal Health Research Centre, St Mary's Hospital, Central Manchester University Hospitals NHS Foundation Trust, Manchester Academic Health Science Centre, Manchester, UK

**Keywords:** mathematical modelling, placental development, placental shape, spiral artery, stochastic dynamics

## Abstract

In the human, placental structure is closely related to placental function and consequent pregnancy outcome. Studies have noted abnormal placental shape in small-for-gestational-age infants which extends to increased lifetime risk of cardiovascular disease. The origins and determinants of placental shape are incompletely understood and are difficult to study *in vivo*. In this paper, we model the early development of the human placenta, based on the hypothesis that this is driven by a chemoattractant effect emanating from proximal spiral arteries in the decidua. We derive and explore a two-dimensional stochastic model, and investigate the effects of loss of spiral arteries in regions near to the cord insertion on the shape of the placenta. This model demonstrates that disruption of spiral arteries can exert profound effects on placental shape, particularly if this is close to the cord insertion. Thus, placental shape reflects the underlying maternal vascular bed. Abnormal placental shape may reflect an abnormal uterine environment, predisposing to pregnancy complications. Through statistical analysis of model placentas, we are able to characterize the probability that a given placenta grew in a disrupted environment, and even able to distinguish between different disruptions.

## 1. Introduction

Pregnancy outcome is fundamentally dependent on placental function, which is in turn related to placental shape and structure. Epidemiological data suggest that alterations of placental shape may be increased in pregnancies with adverse outcomes, including low birthweight pregnancies [1–3], and in preeclampsia [4]. The effect of this on the fetus can extend to increased lifetime risk of hypertension and coronary heart disease [5,6]. Placental shape is hypothesized to be determined in early pregnancy when the chorion, which covers the spherical embryo in three dimensions, regresses to form the chorion laeve and the placenta [7]. The underlying determinants of the relationship between placental shape and function are not fully understood.

In common with other events occurring in early-to-mid pregnancy, determination of placental shape is difficult to study *in vivo* and *ex vivo* owing to the limits of imaging, availability of tissue and if tissue is obtained, then the ultimate outcome of the pregnancy remains unknown. To address these difficulties, several groups have applied mathematical modelling techniques to the placenta including: oxygen transfer across the villus [8], development of placental shape [9] and fetoplacental blood flow [10]. With relevance to placental shape, Yampolsky *et al*. [9] used a diffusion-limited aggregation (DLA) model to general placental vascular trees; using this model, different shapes could be generated. To further investigate the hypothesis that critical elements of placental shape are determined by the uterine environment in early pregnancy, we aimed to develop a mathematical model that included entry of nutrients/oxygen from the maternal epithelium during early placental villous development and subsequent vascularization. We then aimed to run this model to determine the effects of changing the maternal environment on placental shape.

## 2. Placental growth model

We assume that placental villous development and subsequent angiogenesis is a chemotactically driven process, with the attracting chemical X being maximally available to growing placental tissue once it reaches the opening of a spiral artery; potential chemoattractant(s) released from maternal spiral arteries include oxygen, growth factors, cytokines or nutrients [11]. For the purposes of this paper, which focuses on the initial exploration of a model, we have modelled a single chemoattractant factor that determines placental growth, and thus placental morphology. We have selected oxygen as the potential chemoattractant, as this is known to be a potent regulator of angiogenesis explored in other *in silico* models [12]. We do not claim to know the growth factor(s) in reality, but have used oxygen as a reasonable hypothetical example, because diffusion rates in human tissue are well documented. We have aimed to model the formation of the placenta from approximately four weeks gestation when distinct villi are present on the surface of the chorionic sac (1–2 mm long), until the end of the 12th week of gestation when the chorion laeve is formed [13]. We have stopped the model at this point, because the position of the placenta within the uterus overlying the spiral arteries is now mostly determined; as the pregnancy progresses, the placenta will continue to grow and develop but will occupy the same part of the uterine surface.

### 2.1. Dynamics of stem villous growth

We model the growth of the fetal villous tree by a stochastic differential equation. We assume that the direction of growth of the stem villi is dominated by the chemoattractant emanating from the maternal spiral arteries, which are fixed points in the decidua. Because the stem villi's detection of the gradient of the chemoattractant is likely to be noisy, we model this through the addition of Brownian motion to the trajectory. Therefore, the villous tip 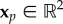 is acting in an evolving potential *V*(**x**, *t*), owing to the concentration of chemoattractant.

The potential itself is evolving for two reasons. The first is that we assume that the tips of stem villi are themselves sinks for the chemoattractant, at a rate comparable to that of an activated spiral artery. Because all the other stem villous tips in the system are moving, the potential function itself is constantly changing. The second is that we assume that all the spiral arteries are initially ‘switched off’. A spiral artery only becomes ‘switched on’ if a stem villous tip comes within a given radius of that artery. We assume that within this radius, the trophoblast emanating from the placenta will reach the spiral artery and prepare it for inclusion in the maternal uteroplacental vascular network [14]. Once this has happened, we assume that the spiral artery has become a source of the chemoattractant. We describe the exact form of the potential function *V*(**x**, *t*) in §3.

Taking into account the effect of the chemoattractant potential field *V*, and the noisy observation of this potential field by the villous tip, we arrive at the following stochastic differential equation for the evolution of the position of the villous tip:2.1
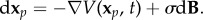
Here **B** is a two-dimensional Brownian motion, and *σ* is the standard deviation of the noise in the trajectory of the villous tip, owing to noisy observations of the chemoattractant gradient. The noise is isotropic, i.e. its variance is the same in all directions. Note that trajectories of this system are attracted to regions where *V* is the lowest. Trajectories of this form can be discretized using the Euler–Maruyama scheme [15], which, for a time step of size Δ*t*, is given by the following expression:2.2
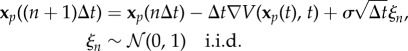
We use formula (2.2) for the update of each of the stem villous tips in the system, and record the history of the trajectories of each of the tips. The path of the tip represents the shape of the villous tree being grown. Higher-order numerical methods exist for the solution of stochastic differential equations, such as the Milstein method [16], or stochastic analogues of higher-order Runge–Kutta methods [15]. However, for our needs, the Euler–Maruyama scheme is sufficient.

### 2.2. Branching of the fetal villous tree

Based on knowledge of the potential field, we propose a branching condition for the growth of the villous tree. Neglecting some corrections which will be given in more detail below, we assume that branching occurs when the gradient of the potential field has a small absolute value at the point that the villous tip is occupying. Low gradient means poor information for the villous tip to decide in which direction it should grow. If the concentration of chemoattractant is simultaneously non-zero in this location, then this small gradient will, in general, be due to the villous tip's path heading in a direction that would approximately perpendicularly bisect a line between two spiral arteries. In this situation, it is advantageous for the villous tree to split in order to attach itself to both spiral arteries.

We also add further corrections to prevent branching near the cord insertion (where it is very likely that there will be several sources of chemoattractant of similar strength per distance), further where the chemoattractant concentration is essentially zero in that local area, at the sources (except the initial stages of angiogenesis), or near another tip of a growing villous tip. Below, we discuss all these conditions in more detail.

We start with an initial number *N*_0_ = 4 of growing tips from the point of insertion of the umbilical cord on the placental disc. The probability of branching per time unit is proposed to be in the following form:2.3
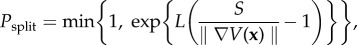
meaning that the flatter the potential field is (compared with the value of parameter *S*) at a given point **x**, the (exponentially) more probable the branching is. The parameter *L* represents a likelihood constant of branching. A few corrections are needed though as the potential field contains contributions from sources (negative) and growing tips (positive). The considered corrections for branching probability at the growing tip **x***_p_* are the following:

— If *V*(**x***_p_*) > 0, then *P*_split_ = 0. This condition represent a no-split requirement if the concentration of chemoattractant is essentially zero in that local area as *V*(**x***_p_*) > 0 means that there is more consumption of the chemoattractant by the growing tips than is being produced by the sources in that area.
— If *||***x***_p_*|| < *ε*, then *P*_split_ = 0, for some 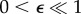. We prevent branching from happening when the tip **x***_p_* is close to the umbilical cord insertion as the gradient there can be expected to be low owing to several sources of similar magnitude distributed around the insertion. In general, a large amount of branching of the villous tree at the cord insertion is not usually observed in real placentas.
— If distance to the nearest source is less than *ε* and the nearest source has not been reached yet by any growing villous tips, then the probability is set to zero *P*_split_ = 0. Only in initial stages of angiogenesis do we assume that branching occurs once a source is reached (until some number *N*_ini_ = 6 of sources is reached). Otherwise, growing tips would quickly find some nearest sources without branching enough to enable a full villous tree with *N*_T_ = 30 terminations in sources to be grown.
— If distance to the nearest growing villous tip is less than *ɛ*, then we set the probability of branching to zero *P*_split_ = 0 to prevent multiple branching of the same villous tip in a very short time.

### 2.3. Activation of spiral arteries

As described before, we assume that at the time when stem villi are formed, and proximate to the decidua, the spiral arteries are not releasing significant amounts of the chemoattractant factors. They only become ‘switched on’ when in close enough proximity to a villous tip, in order for the trophoblast to invade the site [14]. Therefore, we define a parameter *R*_troph_ > 0. After updating the positions of the villous tips, all of the ‘switched off’ spiral arteries are checked to see if there are any villous tips within a distance *R*_troph_ of them. If they are, then the spiral artery is ‘switched on’, and its effect is added to the potential function for the next time step (see §3 for more details of the potential function).

A spiral artery only becomes inactive again if a villous tip is assumed to have terminated at this source, as we describe in §2.4.

### 2.4. Stopping condition for stem villous tips

As simulated angiogenesis is dimensionless, with one unit equal to a typical distance between spiral arteries (12 mm, see §3.2), we include this scaling in our potential field. Further, because both the potential and its gradient are growing to infinity as the source is approached and since, as was mentioned above, in the vicinity of a source there are two villous tips that are actually growing towards each other (one from villous tree and one being the villous tip itself), we stop the growth of a villous tip once it is closer than a certain threshold value *D*_stop_ to a source and consider it to have reached that source. At this point, we assume that the sink of the villous tip and the source of the spiral artery cancel each other out, and both are turned off for the remainder of the simulation.

### 2.5. Stopping condition for the villous tree

It has been observed that each placenta is formed from 30 to 60 lobules (also termed placentones or cotyledons) [17,18]. For the purposes of this model, we assume that there is one spiral artery opening per placental lobule [19]. We decided to choose a number of arteries *N*_T_ that are reached before the simulation finishes. As soon as this threshold has been reached, the growth of the villous tree is stopped, and any parts of the tree which have not yet reached a spiral artery are removed from the simulation (they have not found a source of nutrition and therefore will simply not develop into a fully functioning part of the villous network). In this model, we assume that once this process is complete, all villi are subsequently vascularized, and then link up with the vascular tree developing on the chorionic plate.

## 3. Finding an appropriate potential field

We propose a potential field of the growth factor that a single spiral artery produces to correspond to a diffusion of a chemical from a point source with a magnitude *M* (neglecting the possible effects of spiral artery size) constant in time from its onset at time *t*_0_. Thus, the potential field *V*(**x**, *t*) is a solution of3.1
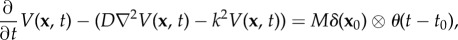
where consumption of the attracting chemical by surrounding tissue, with a rate *k*^2^, is considered and *θ*(*t*) denotes a Heaviside function. Consumption of oxygen is more appropriately modelled by a Michaelis–Menten term instead of linear consumption, but this dependence is rational when oxygen concentration is low: *M*_0_*P*/(*P*_0_ + *P*) ≈ (*M*_0_/*P*_0_)*P*. The solution, in general, to this problem can be found by calculating Green's function (also called the fundamental solution) of the differential operator using Fourier transformation. However, the potential with its gradient (needed for chemotactic growth) cannot be rewritten into analytical or closed form which is desired for our purposes. That is why we will look for a steady-state solution instead which essentially means that we assume the diffusion process of the attracting chemical *X* to be much faster compared with villous tree growth. It can be shown (see the electronic supplementary material for derivation) that the stationary potential field *V*(**x**) is proportional to the modified Bessel function and is given by3.2
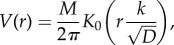
with asymptotic behaviour 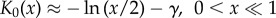.

### 3.1. Initial distribution of the spiral arteries

The initial distribution of the spiral arteries that we have implemented can be described by the following algorithm:

(1) It is believed that there are roughly 200 spiral arteries in an average uterus. We take it to be exactly *N*_sources_ = 200. We assume that as the fetus grows, and the uterus distends, the distance between the spiral arteries increases in proportion to increase in placental size.
(2) We randomly distribute this number of spiral arteries throughout the considered circular domain which we assume has radius 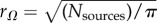. Therefore, on average, there is one source per 1 unit squared.
(3) Create random samples from the uniform distribution of sources on the circle, conditioned on no two sources being closer than *ρ*_min_ = 0.7 to each other. Note that a uniform distribution on a circle is obtained by sampling 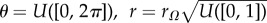, and taking **x** = *r*cos(*θ*).

We hypothesize that disrupted placental shape may be related to blocked, damaged or missing spiral arteries in certain regions of the uterus. We also consider the effect of maldevelopment of the maternal vascular network leading to a whole region of blocked/switched off spiral arteries. Low and irregular densities of spiral arteries close to the point of adhesion of the conceptus to the uterus can cause, as we show in §6, irregularly and bipartite-shaped placentas, and off-centre cord insertions. This in itself may not be the cause of problems in pregnancy, but we hypothesize that it is certainly an indication of a poor or irregular blood supply to the placenta, and therefore to the fetus, which in turn could cause low birth weight.

The initial distribution of spiral arteries in simulations with disrupted scenarios is carried out in the same way as described above at first. Once a uterus is stocked with 200 spiral arteries as before, we then identify which of these lie within the region where sources (spiral arteries) are being blocked/switched off. We consider this region to be circular with a given radius *r*_disrupt_ and centre *s*_disrupt_ (WLOG to be on the *x*-axis). Any spiral arteries in this region are switched off, and not replaced anywhere else in the domain, leading in general to less than 200 healthy spiral arteries. For the simulation of bipartite placentas, we hypothesize that this is caused by two disrupted regions which are symmetric with respect to the cord insertion.

### 3.2. Parameter values

The overall potential landscape is a sum of point source contributions from each active spiral artery and growing tip. We consider exactly the same potential for a point source but with an opposite sign, because we assume the growing tip to be a constant sink of attracting chemical. As there is not enough data to determine the magnitude of point sources of spiral arteries and compare them with sinks and with each other, we assume them to be of equal magnitude (so once a source is actually reached by a growing villous tip, their potentials cancel out). To summarize, the potential driving the chemotactical angiogenesis is3.3
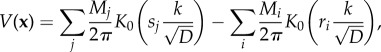
where the second summation is carried out over all sources *i* with magnitudes *M_i_* and *r_i_* being the distance from the given point (**x**) to a source *i*. Similarly, the first summation is carried out over all current non-terminated villous tips with magnitudes *M_j_* and *s_j_* being the distance from the given point (**x**) to a villous tip *j*. Magnitudes of all sources and sinks were considered to be equal, *M_i_* = 1, ∀*i* and *M_j_* = 1, ∀*j*. Note that there is essentially only a single parameter 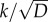 which needs to be determined.

A relevant value for the consumption rate of oxygen in placental tissue was calculated from *in vitro* data to be 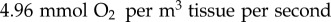 [20]. This leads to a value of *k* = (32/1.429) × 4.96 × 10^−3^ = 0.11 × 10^−3^ s^−1^. Diffusion coefficient in the endometrium is *D* = 5.5 × 10*^−^*^10^ m s^−1^ [21]. Thus, the single model parameter can be estimated to be 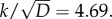


The last task is to estimate the actual distance between spiral arteries in the placenta in this considered phase of the villous tree development. There is a consensus that there are roughly 200 spiral arteries in the whole uterus. The three dimensions of the uterus have been measured in both nulliparous and multiparous women. The mean size of uterus is measured using three quantities: length is measured from the fundus to the external os, the anterposterior diameter is the maximum length in the midsagittal section of the body of the uterus in the anterposterior direction, and uterine width is the maximum measurement obtained in a cross section of the fundus.

Let us assume for the sake of simplicity that the uterus is of ellipsoidal shape in early stages of pregnancy. The formula used to compute the surface area of an ellipsoid is an approximation known as Knud Thomson's formula with *p* = 1.6075 [22]:
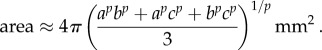


The mean values reported in the literature vary [23–27], but they all lead to estimation of distance 12–13 mm between spiral arteries, where we have used the above mentioned Knud Thomson's formula. We shall consider the mean uterus size to be 75 × 25 × 50 mm [27], with the estimate of 12 mm being the average distance between spiral arteries (table 1).

**Table 1.** List of parameters.

parameter value	meaning
Δ*t* = 10*^−^*^3^	time step used in the Euler–Maruyama scheme for villous tree growth
*L* = 0.1	likelihood constant of branching
*S* = 0.5	branching level
*ɛ* = 10*^−^*^2^	used in corrections of probability branching condition
*D*_stop_ = 10*^−^*^2^	threshold value for stopping near sources
*R*_troph_ = 1.5	radius of effect of trophoblast from villous tips
*N*_0_ = 4	initial number of growing tips from cord
*N*_ini_ = 6	initial phase of angiogenesis when we consider a split to occur each time a source is reached (until *N*_ini_ sources were reached)
*N*_T_ = 30	number of arteries that are reached before the simulation finishes
*ρ* = 5	a scaling constant used for finding suitable resolution/magnitude of ‘an oxygen supply for placenta tissue from its vascular network’
*σ*_thresh_ = 0.001	threshold value for obtaining placental shape

## 4. Estimating placental shape

**Figure 1.**
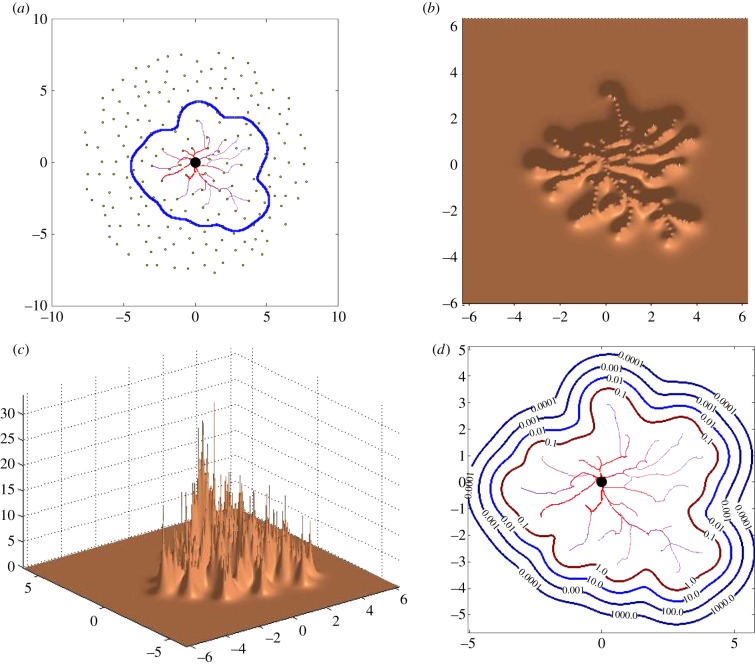
An example of shape estimation from a landscape of oxygen supply for placenta from its vascular network. (*a*) A given network generated by the presented algorithm together with placental shape estimation. (*b*,*c*) Plot of OxLevel(*x*) function: height of the surface represents oxygen density over the two-dimensional network. (*d*) Contour plot of OxLevel(*x*) function for several values of *σ*_thresh_. (Online version in colour.)

To estimate the shape of a given placenta based on its villous tree, we use the following approach. Given a point in space (in the plane), we simply ask whether it is inside the considered placenta or not. We count a sum of weighted distances of the considered point to each segment (of a given length) of the villous tree, and if it is less than a certain threshold value *σ*_thresh_, then we regard the considered point to be within the placenta. This choice is motivated by diffusion from the vascular network, where we assume that concentration of a chemical from a constant source decays with distance *x* as a modified Bessel function of the second kind *K*_0_(*x*) (see §3). We also assume that a tissue (at the considered point) can survive only once it has access to a certain necessary (or minimal) amount of oxygen. Then, to calculate what the level of oxygen is at the given point, we need to sum the contributions from the whole vascular network, as we consider it to be the only source of oxygen. To summarize
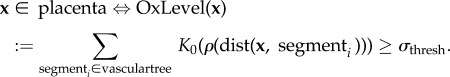
The scaling constant *ρ* is used for finding suitable resolution/magnitude of ‘an oxygen supply for placenta from its vascular network’ (figure 1*b,c*). The length of all segments is the same and chosen to be 0.05, and the threshold value was set to *σ*_thresh_ = 0.001 which is used for obtaining a contour of the placenta (figure 1*d*).

## 5. Visualization of the placenta

Here, we describe how we visualize the output of the model as described in the previous sections. In the visualization of the output of the model, we had two aims.

— To create images of the vascular tree which were as realistic as possible, in order to visually compare them with the vascular trees of real placentas.
— To create images from the output of the model to indicate the shape of the placenta, and the position of the cord insertion, in order that we can apply the statistical measures as described in our previous work [28].

**Figure 2.**
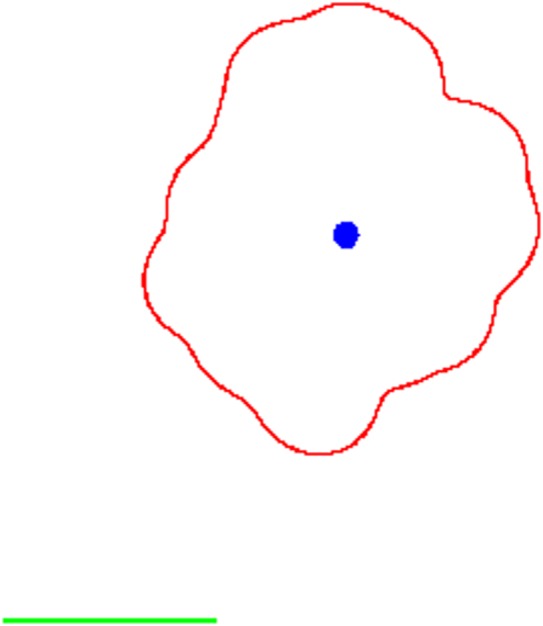
An example of the output of the model for analysis of the shape and cord insertion position of the placenta. (Online version in colour.)

The second objective is easily achieved, simply by plotting a circle at the point of cord insertion, an outline of the placental tissue, and a scale bar (figure 2).

To achieve a more realistic look for the plotting of the vascularized villous tree, we also wanted to visualize the relative thickness of the branches of the villous tree. When two villous branches meet to form one larger branch, we can make an assumption about the relative thicknesses of the branches based on Murray's law [29]. Therefore, given a pair of branches with widths *w*_1_, *w*_2_ > 0, respectively, the combined branch where they join will be of width5.1
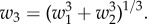


Once we have the villous tree, we first assign widths to each of the villous tips present at the end of the simulation that have terminated at a spiral artery. This width is based on the number of villous tips *n* which have terminated at the same spiral artery. Usually, this will be just one, but on occasion two or more branches can find their way to the same spiral artery. Given that we assume that each spiral artery has width equal to 1 (non-dimensionalized width), we can assume that each of the emanating villous branches has width
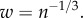
Iterating back down the tree using the relation (5.1), we can assign realistic relative widths to all of the villous tree. We can then add this detail to our plot, to achieve pictures such as shown in §6.

## 6. Numerical results

**Figure 3.**
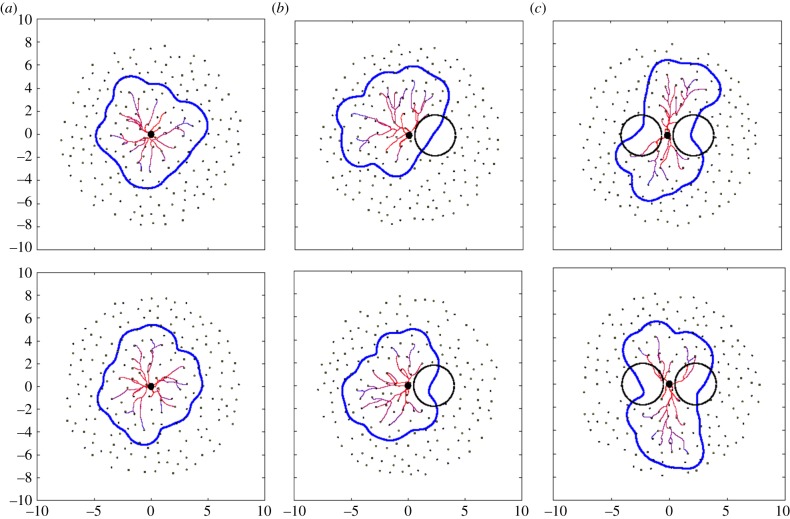
Examples of typical vascular tree predictions in the considered scenarios: (*a*) control, (*b*) disrupted (10 arteries blocked), (*c*) bipartite (20 arteries blocked symmetrically according to cord insertion). The spiral arteries are depicted as black dots, blocked region by a black circle, vascular tree and estimated placental shape by solid lines. (Online version in colour.)

Here, we show results of the above presented model for placental vascular tree growth together with shape estimation. Three qualitatively different scenarios were considered. They differ only in the initial distribution of spiral arteries as described in §3.1. *Control* case corresponds to 200 available spiral arteries representing a ‘healthy’ individual with none of the spiral arteries blocked. In the *disrupted* scenario, a circular region with a radius 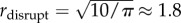 and centre *s*_disrupt_ = 0.5 + *r*_disrupt_ ≈ 2.3 has been blocked. This size of the radius means that on average 10 spiral arteries are blocked. The last considered case was *bipartite* case where two symmetrically positioned regions were considered having the same size and distance from the cord insertion as in the disrupted scenario. Typical plots in these three cases are given in figure 3. Of course, the blocking of spiral arteries will have an impact almost only when a region *close* to the attachment of umbilical cord is affected. This is because diffusion of attractor from spiral arteries (the shape of potential field) is considered to be the main driving force of angiogenesis in placenta.

**Figure 4.**
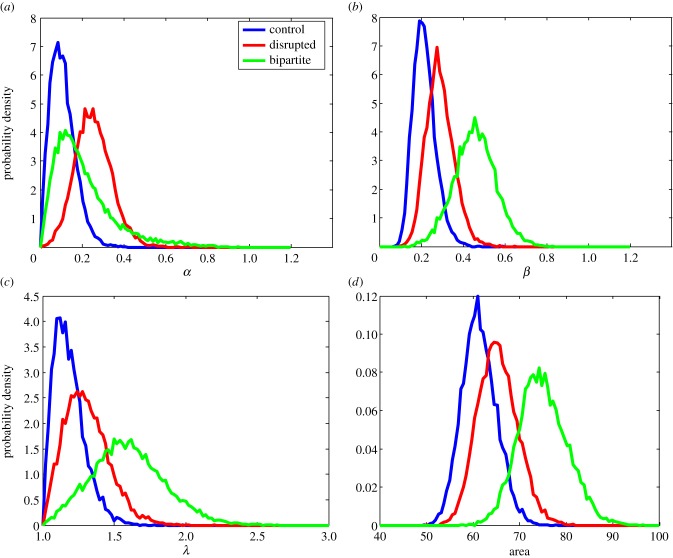
(*a*–*d*) Probability density of proposed measures based on 10 000 simulations of the presented model for placental vascular tree and shape development. See the text for description of the measures. (Online version in colour.)

Individual realizations of the presented stochastic model are not of interest here, because there is indeterminism involved in each example. We are more interested in average behaviour. To draw qualitative conclusions/predictions from the model, we need to statistically analyse a large set of data from the model. For this purpose, we simulated the formation of 10 000 placentas in each scenario, and calculated statistical measures as proposed in our previous work [28] using techniques described in §5 to extract umbilical cord placement within placenta, outer shape of placenta and scale bar. Only these results (plotted in figure 4) can be compared with clinical data (ideally again large set of data). The typical examples shown in figure 3 show expected behaviour.

The used measures have roughly the following meaning (for more detailed descriptions, see our previous work [28]): *α* quantifies how central the cord insertion is, *β* is a measure of circularity (*β* = 0 for a circle), *λ* is the aspect ratio of longer to shorter axis from ellipticity, and we also measure the area of each placenta. These measures might enable us to estimate with a reasonably high probability to which scenario a given placenta most probably corresponds.

Note that centrality corresponds well to control placentas as the average value together with magnitude of variations is clearly the lowest for the undisrupted control case. The model predicts that placental area increases with the severity of disruptions and is likely to be related to necessity for the placenta to reach a given number of sources of growth factor (spiral arteries). Control placentas are the most circular ones, with the bipartite placentas the least circular. Circularity seems to be a better measure to distinguish between disrupted and bipartite cases which is intuitive. Finally, the aspect ratio confirms that control placentas are the most round, and furthermore can be used to further distinguish between the disrupted and bipartite scenarios.

**Figure 5.**
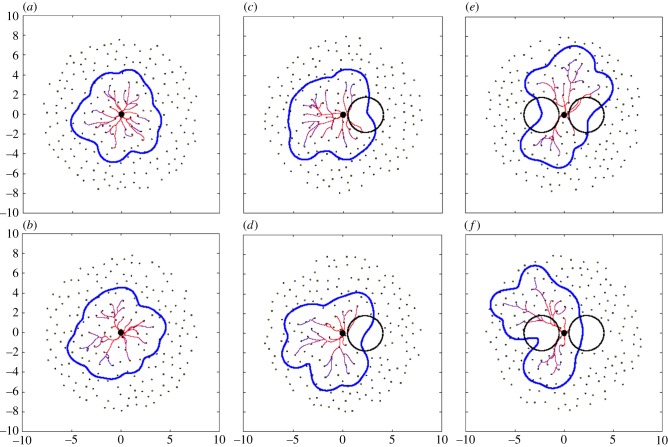
Six randomly chosen examples of placentas were used to estimate their relevance to each scenario using the proposed statistical measures. They are in fact particular realizations of control (*a*,*b*), disrupted (*c*,*d*) and bipartite (*e*,*f*) group. (Online version in colour.)

Let us consider a set of six randomly chosen placentas (two from each scenario) as generated by the presented model (figure 5), and let us use the statistical results from our model to estimate to which scenario a given placenta belongs. It serves as a demonstration of the applicability of the presented model together with the proposed measures but also as a confirmation.

**Figure 6.**
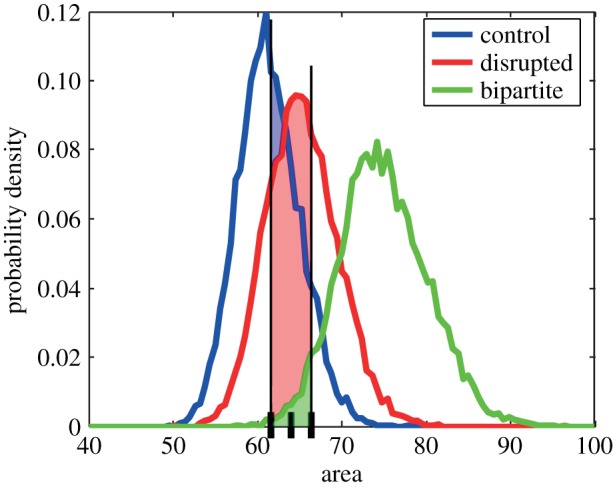
An illustration of probability estimation based on the surface underneath probability distribution function in each scenario (note that regions do overlap). (Online version in colour.)

For example, consider two placenta from figure 5 with values *α* = 0.080, *β* = 0.158, *λ* = 1.032, area = 60.0 (figure 5*a*) and *α* = 0.120, *β* = 0.167, *λ* = 1.154, area = 63.43 (figure 5*b*). Then, we can estimate the probability of each measure that the considered placenta corresponds to control, disrupted or bipartite case by calculating the integral of probability density functions in all cases over a symmetric 1% neighbourhood of the measure relevant scale (figure 6). Finally, by comparing these values, we arrive at probabilities that the considered placenta corresponds to one of the three cases. Percentages are given in table 2. In every example that we looked at, the highest probability computed for the three scenarios correctly identified from which scenario the placenta had come.

**Table 2.** Probabilities of correspondence of placentas from figure 5 to each scenario.

figure 5*a*	control (%)	disrupted (%)	bipartite (%)	figure 5*b*	control (%)	disrupted (%)	bipartite (%)
*α* = 0.080	64	6	30	*α* = 0.120	54	12	34
*β* = 0.158	89	10	1	*β* = 0.167	87	12	1
*λ* = 1.032	76	20	4	*λ* = 1.154	62	32	6
area = 60.0	68	32	0	area = 63.43	47	50	3
avg.	74.25	17	8.75	avg.	62.5	26.5	11
figure 5*c*	control (%)	disrupted (%)	bipartite (%)	figure 5*d*	control (%)	disrupted (%)	bipartite (%)
*α* = 0.260	6	65	29	*α* = 0.312	2	72	26
*β* = 0.217	65	33	2	*β* = 0.275	29	63	8
*λ* = 1.138	62	32	6	*λ* = 1.100	72	23	5
area = 64.19	41	55	4	area = 67.50	22	58	20
avg.	43.5	46.25	10.25	avg.	31.25	54	14.75
figure 5*e*	control (%)	disrupted (%)	bipartite (%)	figure 5*f*	control (%)	disrupted (%)	bipartite (%)
*α* = 0.202	20	46	34	*α* = 0.486	0	35	65
*β* = 0.492	0	3	97	*β* = 0.456	0	6	94
*λ* = 1.718	0	11	89	*λ* = 1.577	3	27	70
area = 72.69	2	21	77	area = 72.04	2	27	71
avg.	5.50	20.25	74.25	avg.	1.25	23.75	75

## 7. Discussion

Owing to the inaccessibility of the placenta during pregnancy, mathematical models have recently been used to study several aspects of placental physiology, including amino acid transport [30], placental shape [9], oxygen transfer across the villus [31], blood flow through the fetal circulation [32,33] and blood flow through the maternal component of the intervillous space [8]. In addition, models have explored pathological states including: fetal blood flow in hypertension [33], abnormal fetal blood flow in twin–twin transfusion syndrome [34] and reduced intervillous perfusion [35]. In these situations, mathematical models have the advantages that they can be based on *ex vivo* and *in vitro* experimental data and, if they include biological variation, can be run many times to reproduce many individual pregnancies.

Here, we developed a model of early placental development based on data regarding the number of spiral arteries in the uterus and within the placental bed, the oxygen consumption of placental tissue and the diffusion of oxygen within the endometrium. We aimed to test the hypothesis that placental size or shape was closely related to presence and distribution of spiral arteries in the vicinity of placental development. Combined with objective measurements of placental shape our model suggests that there is significant variation in normal placental shape with a normal distribution for circularity, ellipticity, aspect ratio and cord insertion; with few placentas being perfectly discoid with a central cord insertion. These findings are identical to descriptions of placental shape and cord insertion in a cohort study of 861 infants which lends biological validity to our model [36]. In our model, disruption of the normal field of spiral arteries significantly alters placental shape, resulting in a greater frequency of lateral cord insertion and bipartite placentas. This observation supports the hypothesis that maternal vascular bed determines placental shape to some extent. Thus, the abnormal placental shape, cord insertion and reduced placental area observed in small-for-gestational-age infants may result from an abnormal uterine environment [37,38], rather than abnormal placental shape being an independent determinant of fetal growth. Critically, we are not suggesting that all abnormal placental shapes are derived in this way, but that this is a biologically and mathematically plausible explanation. It is possible that other disruptions of the decidual environment such as uterine scars (myomectomy or caesarean section) could alter the chemoattractant profile which could also adversely affect placental shape. These potential disruptors of the decidual environment could also be added into development of this mathematical model.

The strengths of this model include that it is based on *ex vivo* and *in vitro* observations and it has a random component which mimics biological variation assessed by formulae which have been successfully used to evaluate placentas from normal pregnancies and those with small-for-gestational-age infants [28]. In addition, this model was developed by a multidisciplinary team, providing biological, clinical and mathematical input. The images of the placental vasculature that were produced by this model are consistent with those seen in human biology [39].

However, as with all mathematical models, there are limitations. This model assumes that all nutrients, growth factors or oxygen will emanate from the same point source and have similar chemoattractant properties. Although all parameters are crucial to the model, an exhaustive parameter study to quantify the sensitivity of the outcomes of the model to the parameters was not feasible. Furthermore, some of the constants, namely those involved in the calculation of the branching properties, were tuned to the values of other relevant parameters, i.e. the diffusion constant of the chemoattractant. We have been able to show that changing the number of initial branches of the umbilical artery from 4 to 2 results in negligible changes to the outcomes of the model. This can be seen in the electronic supplementary material, where we present analogous results to those in figure 4, where we have changed the initial number of branches *N*_0_ to 2. This reflects the observation that placental shape does not change significantly in the case where there is a single umbilical artery [40]. Some observations, particularly about the number of spiral arteries in a human uterus and placental bed, are from experiments performed over 50 years ago, but these would be almost impossible to repeat owing to technical and ethical issues. Nevertheless, these estimates form the basis of modern understanding of uterine anatomy and changes in spiral arteries in early pregnancy.

There has been one other published study using a mathematical model to derive placental shape from early pregnancy events. Yampolsky *et al*. use a DLA tree to model the development of fetal vascular tree, on which placental shape is superimposed [9]. These images were compared with images of placental vascular trees and placental shape related to fetal weight via a metabolic scaling law [41]. This model also found that non-central cord insertion was associated with lower birthweight infants [3]. Although this model produces vascular trees which are reminiscent of the vascularization of the chorionic plate, it is based on random growth of the vascular tree from a central point (the putative umbilical cord insertion); critically, this assumes that fetoplacental vascularization determines placental shape. However, placental vascularization is thought to be a secondary event occurring within the formed placental villi, with vessels growing out from the umbilical cord insertion, meeting vessels developing within stem villi [17]. Our model suggests that where there are no maternal spiral arteries, there is a reduction in trophic factors, villi do not develop and there is no subsequent fetoplacental vascularization. Thus, placental shape and the pattern of vessels on the chorionic plate may not be a purely random event, but is potentially determined by the pattern of maternal spiral arteries.

The invasion and conversion of spiral arteries in the maternal uterus by the placentally derived extravillous trophoblast has been likened to invasion and growth of malignant tumours [42,43]. Interestingly, this is another area where mathematical modelling has been used to understand the interplay between vascularization and consequent tumour growth to produce a powerful tool that can explain the *in vivo* dynamics of tumours [12]. Extension of our mathematical model to include the conversion of maternal vasculature by extravillous trophoblast may provide further understanding of the critical events in early pregnancy which determine placental size, shape and ultimately pregnancy outcome.

The aim of this work was to understand whether variation in placental shape might relate to the uterine milleu in an attempt to understand why changes in placental shape are reported more frequently in pregnancy complications. Imaging technology is currently improving so that placental shape, size, cord insertion and vascularization can be determined by ultrasound or magnetic resonance imaging. Therefore, it is conceivable that assessment of placental size and shape could be carried out *in vivo* at any point after 12 weeks gestation and used to identify pregnancies which are at risk of poor pregnancy outcome in later pregnancy. We recognize that placental measurements will not remain absolute, but interestingly the best correlations between ultrasound scan at 11–14 weeks and placental appearances at term were cord eccentricity (*r*^2^ is −0.63) and cord centricity (*r*^2^ is 0.61) [44], suggesting that placental shape can be evaluated at that point. However, further mathematical modelling would be needed to explore the changes in placental size and shape as pregnancy progresses to inform clinical studies.

## Supplementary Material

Supplemental File
