# Supplementary material for: A stochastic model for early placental development
Source: J R Soc Interface. 2014 Aug 6;11(97):20140149. doi: 10.1098/rsif.2014.0149 (PMC4208356; doi:10.1098/rsif.2014.0149)
Supplement: Supplemental File [file rsif20140149supp1.pdf]

A Stochastic Model for Early Placental Development: Supplemental File

By Simon Cotter, Vaclav Klika, Laura Kimpton, Sally Collins and Alexander Heazell

Estimation of potential landscape due to a constant source independent of time

In this section, we shall estimate the potential field of the growth factor that a single spiral artery produces to correspond to a diffusion of a chemical from a point source with a magnitude M (neglecting the possible effects of spiral artery size) constant in time from its onset at time t_0. Thus the potential field $V(\mathbf{x}, t)$ is a solution of:

$$\frac{\partial}{\partial t}V(\mathbf{x},t) - \big(D\nabla^2 V(\mathbf{x},t) - k^2 V(\mathbf{x},t)\big) = M\delta(\mathbf{x}_0) \otimes \theta(t-t_0) \tag{0.1}$$

where consumption of the attracting chemical by surrounding tissue, with a rate k^2, is considered and $\theta(t)$ denotes a Heaviside function. Consumption of oxygen is more appropriately modelled by a Michaelis-Menten term instead of linear consumption but this dependence is rational when oxygen concentration is low: $\frac{M_0 P}{P_0+P} \approx \frac{M_0}{P_0}P$.

We shall find the Green's function for the operator on the left hand side of (0.1), can be obtained using Fourier transformations, and is given by the following expression:

$$\varepsilon(\mathbf{x},t) = \theta(t)\frac{1}{4\pi Dt}\exp\left(-\frac{\|\mathbf{x}\|^2}{4Dt} - k^2 t\right), \quad \mathbf{x} = (x_1, x_2),\ \|\mathbf{x}\| = \sqrt{x_1^2 + x_2^2}. \tag{0.2}$$

Thus the potential V satisfying equation (0.1) can be obtained using this result, and is given by:

$$\begin{aligned} V(\mathbf{x},t) &= \varepsilon(\mathbf{x},t) * \Big(\delta\big(\mathbf{x}-\mathbf{x}_0\big) \otimes \theta(t-t_0)\Big) \\ &= \varepsilon\big(\mathbf{x}-\mathbf{x}_0, t\big) * \theta(t-t_0) \\ &= \theta(t)\int_0^t \varepsilon\big(\mathbf{x}-\mathbf{x}_0, \tau - t_0\big)\mathrm{d}\tau, \end{aligned} \tag{0.3}$$

where the operator $*$ denotes convolution in generalised functions (or distribution). However, the potential with its gradient (needed for chemotactic growth) from equation (0.3) cannot be rewritten into analytical or closed form which is desired for our purposes. That is why we will look for a steady state solution instead which essentially means that we assume the diffusion process of the attracting chemical X to be much faster compared to vascular tree growth. Then the potential $V(\mathbf{x}, t)$ satisfies:

$$-D\nabla^2 V(\mathbf{x},t) + k^2 V(\mathbf{x},t) = M\delta(\mathbf{x}) \tag{0.4}$$

which can be solved in generalised functions $\mathscr{D}'$ as follows. Assuming the angiogenesis process in placental development is planar, one can rewrite the above equation (0.4) into polar coordinates, and taking into account the symmetry of the problem we have

$$-D\left(\frac{\partial^2}{\partial r^2}+\frac{1}{r}\frac{\partial}{\partial r}\right)V(r)+k^2V(r)=M\delta(r). \qquad (0.5)$$

Instead of solving equation (0.4) or (0.5) directly, we will find a solution to the same equation but dropping the point source in the origin

$$-D\left(\frac{\partial^2}{\partial r^2}+\frac{1}{r}\frac{\partial}{\partial r}\right)V(r)+k^2V(r)=0 \qquad (0.6)$$

and such that grows to infinity in the origin and decays with r as it goes to infinity (boundary condition). If we rescale the independent variable r in (0.6) as $\rho = \frac{kr}{\sqrt{D}}$, we obtain a differential equation that can be solved by using modified Bessel functions, as the parameter k^2 is positive. From the considered boundary conditions (potential decays to zero at infinite distance from a given source) we have

$$V(r)=B_0K_0\left(r\frac{k}{\sqrt{D}}\right) \qquad (0.7)$$

with $K_0(x)$ representing the modified Bessel function of the second kind. To relate the magnitude M of the considered point source to the above solution in terms of Bessel functions (determining the constant B_0 based on a value of M) we have to carry out some calculations which are outlined below. One has to choose appropriate test function $\varphi(x,y)\in\mathscr{D}(\mathbb{R}^2)=C_c^\infty(\mathbb{R}^2)$ (test functions are smooth with compact support).

We look for a relation between constants B_0 and M such that $V(\mathbf{x})=B_0K_0\left(\|\mathbf{x}\|\frac{k}{\sqrt{D}}\right)$ is a solution of the reaction-diffusion equation with a point source with magnitude M which we are concerned with for our purposes:

$$M\delta(\mathbf{x})= -D\nabla^2V(\mathbf{x})+k^2V(\mathbf{x}).$$

This is an equality in the space of generalised functions which means that

$$\big(M\delta(\mathbf{x}),\varphi(\mathbf{x})\big)=\big(-D\nabla^2V(\mathbf{x})+k^2V(\mathbf{x}),\varphi(\mathbf{x})\big),\quad \varphi\in\mathscr{D}(\mathbb{R}^2).$$

To obtain the required relation it is enough to consider the following set of test functions

$$\varphi_{\varepsilon,\delta}(\mathbf{x})=\omega_{\varepsilon/2}(\mathbf{x})*\chi_{G_\varepsilon}(\mathbf{x}),$$

where the domain is chosen to be $G=\langle-\varepsilon+3\delta/2,\varepsilon-3\delta/2\rangle\times\langle-\varepsilon+3\delta/2,\varepsilon-3\delta/2\rangle$ and the function ω_ε is defined as follows

$$\omega_\varepsilon(\mathbf{x})=\begin{cases} C_\varepsilon \mathrm{e}^{-\frac{\varepsilon^2}{\varepsilon^2-\|\mathbf{x}\|^2}} & \dots\ \|\mathbf{x}\|<\varepsilon \\ 0 & \dots\ \text{otherwise,}\end{cases} \qquad (0.8)$$

where C_ε is opted so that $\int_{\mathbb{R}^2}\omega_\varepsilon(\mathbf{x})\mathrm{d}\mathbf{x}=1$. Further χ_G is a characteristic function of a domain G and G_ε denotes an ε-neighbourhood of the domain G, i.e.

$$\chi_{G_\varepsilon}(\mathbf{x})=\begin{cases} 1 & \dots\ \mathrm{dist}(G,\mathbf{x})<\varepsilon \\ 0 & \dots\ \text{otherwise,}\end{cases} \qquad (0.9)$$

Note that δ is dependent on ε as $\delta < \varepsilon$ and one can easily check that really $\varphi_{\varepsilon,\delta}$ is a test function for each $\varepsilon > 0$ and $0 < \delta < \varepsilon$. Examples of $\varphi_{\varepsilon,\delta}$ for $\varepsilon = 1$ for several δ values are given in Fig 1.

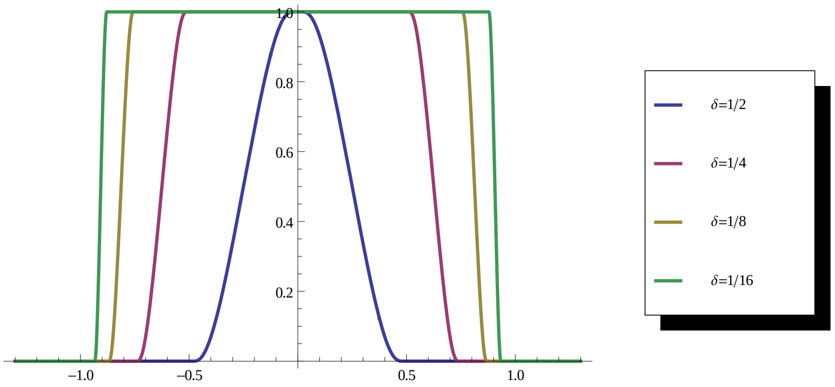

Figure 1. Examples of $\varphi_{\varepsilon,\delta}$ for $\varepsilon = 1$ in one dimension for several δ values. One can observe the convergence towards characteristic function of the interval $\langle -1, 1\rangle$ and notice the declared smoothness of these functions.

Now we calculate each term in the equation when considering only the above mentioned set of test functions $\varphi_{\varepsilon\delta}$

$$\begin{aligned} \underbrace{\lim_{\varepsilon\to 0}\lim_{\delta\to 0}(M\delta, \varphi_{\varepsilon\delta})}_{\text{A}} &= \lim_{\varepsilon\to 0}\lim_{\delta\to 0}(-D\nabla^2 V + k^2 V, \varphi_{\varepsilon\delta}) \\ &= \underbrace{\lim_{\varepsilon\to 0}\lim_{\delta\to 0}(-D\nabla^2 V, \varphi_{\varepsilon\delta})}_{\text{B}} + \underbrace{\lim_{\varepsilon\to 0}\lim_{\delta\to 0}(k^2 V, \varphi_{\varepsilon\delta})}_{\text{C}}. \end{aligned} \quad (0.10)$$

The term A in (0.10) can be easily calculated as follows:

$$A = \lim_{\varepsilon\to 0}\lim_{\delta\to 0}(M\delta, \varphi_{\varepsilon\delta}) = \lim_{\varepsilon\to 0}\lim_{\delta\to 0} M\varphi_{\varepsilon\delta}(0,0) = M,$$

as $\varphi_{\varepsilon\delta}(0,0) = 1,\ \forall\varepsilon, \delta$.

The third term C converges to zero. This can be shown for example by following reasoning (notice that it is shown that function f is locally Lebesgue integrable $f \in \mathrm{L}^1_{loc}$ which proves the first equality):

$$\begin{aligned} |k^2(f, \varphi_{\varepsilon,\delta})| &= k^2 \left| \int_{\mathbb{R}^2} f(\mathbf{x})\varphi_{\varepsilon,\delta}(\mathbf{x})\mathrm{d}\mathbf{x} \right| \\ &= k^2 \left| \int_{B(0,\varepsilon)} f(\mathbf{x})\varphi_{\varepsilon,\delta}(\mathbf{x})\mathrm{d}\mathbf{x} \right| \le k^2 \int_{B(0,\varepsilon)} |f(\mathbf{x})|\,\mathrm{d}\mathbf{x} \\ &= k^2 \int_0^{2\pi}\int_0^{\varepsilon} |rB_0K_0(r)|\,\mathrm{d}r\mathrm{d}\vartheta, \end{aligned}$$

where we used the fact that $0 \le \varphi_{\varepsilon,\delta}(\mathbf{x}) \le 1$. If we consider ε small enough, we can use asymptotic expansion of the modified Bessel function $K_0(x) \approx -\ln(x/2) -$

$\gamma,\ 0 < x \ll 1$ which yields

$$k^2 \int_0^{2\pi} \int_0^{\varepsilon} |rB_0K_0(r)|\,\mathrm{d}r\mathrm{d}\vartheta \approx 2\pi \int_0^{\varepsilon} r(-\ln(r/2) - \gamma)\mathrm{d}r$$
$$= \frac{\pi}{2}\varepsilon^2 \left(1 - 2\mu + \ln\left(\frac{4}{\varepsilon^2}\right)\right).$$

Finally, carrying out limits for $\delta \to 0$ and $\varepsilon \to 0$ we prove that $C = 0$:

$$|C| = \lim_{\varepsilon\to 0}\lim_{\delta\to 0} k^2|(f,\varphi_{\varepsilon,\delta})| \le \frac{\pi}{2}\varepsilon^2\left(1 - 2\mu + \ln\left(\frac{4}{\varepsilon^2}\right)\right) = 0 \qquad (0.11)$$

and that $f(x,y) = B_0K_0\left(\sqrt{x^2+y^2}\right) \in \mathrm{L}^1_{loc}$.

Only the second term B will be calculated as a limit in δ and ε. From the following calculations it will become apparent that $\nabla^2 f(\mathbf{x}) \in \mathrm{L}^1_{loc}$ which means that

$$(\nabla^2 f, \varphi_{\varepsilon,\delta}) = \int_{B(0,\varepsilon)} \nabla^2 f(x,y)\varphi_{\varepsilon,\delta}(\mathbf{x})\mathrm{d}\mathbf{x}.$$

Firstly, we will calculate the inner limit in $\delta \to 0$:

$$\lim_{\delta\to 0}\int_{B(0,\varepsilon)} \nabla^2 f(\mathbf{x})\varphi_{\varepsilon,\delta}(\mathbf{x})\mathrm{d}\mathbf{x} = \int_{B(0,\varepsilon)} \nabla^2 f(\mathbf{x}) \lim_{\delta\Rightarrow 0}\varphi_{\varepsilon,\delta}(\mathbf{x})\mathrm{d}\mathbf{x}$$
$$= \int_{B(0,\varepsilon)} \nabla^2 f(\mathbf{x})\mathrm{d}\mathbf{x},$$

since $|\varphi_{\varepsilon,\delta}(\mathbf{x})| \le \chi_{\langle-\varepsilon,\varepsilon\rangle\times\langle-\varepsilon,\varepsilon\rangle}(\mathbf{x}) \in \mathrm{L}^2(\mathbb{R}^2)$, where $\chi_{\langle a,b\rangle}$ denotes a characteristic function of interval $\langle a,b\rangle$. Then divergence theorem leads to

$$\int_{B(0,\varepsilon)} \nabla^2 f(\mathbf{x})\mathrm{d}\mathbf{x} = \int_{S(0,\varepsilon)} \nabla f(\mathbf{x}).\mathbf{n}\mathrm{d}s = \int_0^{2\pi} \varepsilon \left.\frac{\partial f}{\partial n}\right|_{r=\varepsilon} \mathrm{d}r.$$

Due to symmetry of the problem one can easily deduce that $\left.\frac{\partial f}{\partial n}\right|_{r=\varepsilon} = \left.\frac{\partial f}{\partial r}\right|_{r=\varepsilon} = B_0\frac{k}{\sqrt{D}}K_0'\left(r\frac{k}{\sqrt{D}}\right) = -B_0\frac{k}{\sqrt{D}}K_1\left(r\frac{k}{\sqrt{D}}\right)$ where the recurrence relation for modified Bessel function $\frac{\mathrm{d}}{\mathrm{d}x}K_0(\alpha x) = -\alpha K_1(\alpha x)$ was used. Finally, we will use asymptotic expansion for modified Bessel function $K_1(r) \approx \frac{\Gamma(1)}{2}\left(\frac{2}{r}\right)^1 = \frac{1}{r},\ 0 < r \ll 1$ and thus

$$B = \lim_{\epsilon\Rightarrow 0}\int_0^{2\pi} \varepsilon\left.\frac{\partial f}{\partial n}\right|_{r=\varepsilon} \mathrm{d}r = \varepsilon 2\pi B_0\frac{k}{\sqrt{D}}K_1\left(\varepsilon\frac{k}{\sqrt{D}}\right) \approx 2\pi B_0.$$

We can conclude with a relation between the magnitude M of the point source and the coefficient in front of Bessel function B_0:

$$M = A = B + C = 2\pi B_0 + 0 = 2\pi B_0,$$

or preferably

$$B_0 = \frac{M}{2\pi}. \qquad (0.12)$$
